# Supplementary material for: Conversion of dietary inositol into propionate and acetate by commensal Anaerostipes associates with host health
Source: Nat Commun. 2021 Aug 10;12:4798. doi: 10.1038/s41467-021-25081-w (PMC8355322; doi:10.1038/s41467-021-25081-w)
Supplement: Supplementary file 1 — Supplementary information [file 41467_2021_25081_MOESM1_ESM.pdf]

Supplementary Information

Conversion of dietary inositol into propionate and acetate by commensal *Anaerostipes* associates with host health

Thi Phuong Nam Bui[1,2]*, Louise Mannerås-Holm[3], Robert Puschmann[4,5], Hao Wu[3,6], Antonio Dario Troise[7], Bart Nijsse[1], Sjef Boeren[8], Fredrik Bäckhed [3,9,10], Dorothea Fiedler [4,5] & Willem M. de Vos [1,11]*

Author Information

Affiliations

1 Laboratory of Microbiology, Wageningen University, Stippeneng 4, 6708 WE Wageningen, The Netherlands
2 Caelus Pharmaceuticals, 3474 KG Zegveld, The Netherlands
3 The Wallenberg Laboratory, Department of Molecular and Clinical Medicine, Institute of Medicine, Sahlgrenska Academy, University of Gothenburg, Gothenburg, Sweden
4 Leibniz-Forschungsinstitut für Molekulare Pharmakologie, Robert-Rössle-Straße 10, 13125 Berlin, Germany
5 Institute of Chemistry, Humboldt-Universität zu Berlin, Brook-Taylor-Straße 2, 12489 Berlin, Germany
6 Human Phenome Institute, Fudan University, 825 Zhangheng Road, Shanghai 201203, China.
7 Proteomics & Mass Spectrometry Laboratory, ISPAAM, National Research Council, 80055, Portici (NA), Italy.
8 Laboratory of Biochemistry, Wageningen University, Stippeneng 4, 6708 WE Wageningen, The Netherlands
9 Novo Nordisk Foundation Center for Basic Metabolic Research, Faculty of Health and Medical Sciences, University of Copenhagen, Copenhagen, Denmark.
10 Region Västra Götaland, Sahlgrenska University Hospital, Department of Clinical Physiology, Gothenburg, Sweden
11 Human Microbiome Research Program, Faculty of Medicine, University of Helsinki, Helsinki, Finland.

*Corresponding authors

Willem M. deVos (willem.devos@wur.nl) & Thi Phuong Nam Bui (nam.bui@wur.nl)

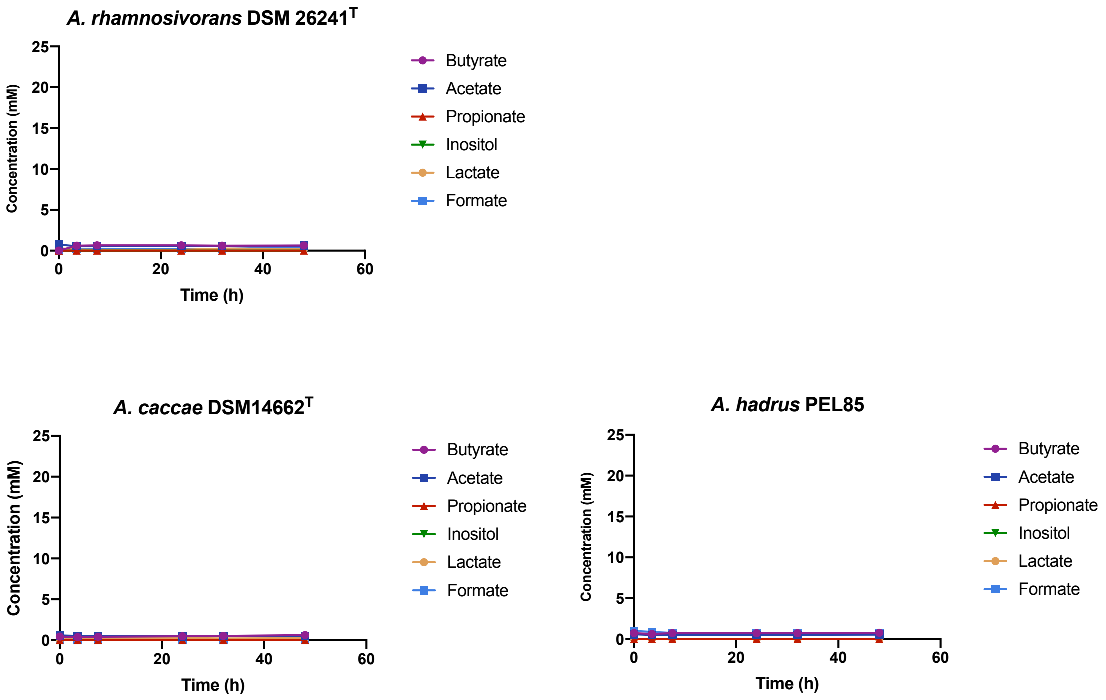

Supplementary Figure 1: Metabolite production by *A. rhamnosivorans* DSM26241^{T}*, A. caccae* DSM14662^{T}*, A. hadrus* PEL85 in bicarbonate buffered medium without *myo*-inositol. The experiments were performed in duplicated and mean values are shown with standard deviation.

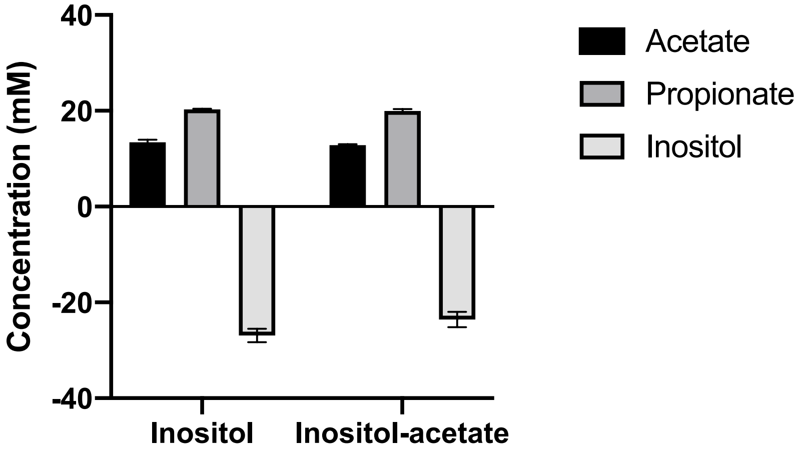

Suppementary Figure 2: Propionate and acetate production by *A. rhamnosivorans* grown on *myo*-inositol or *myo*-inositol plus acetate. Grey and black columns represent produced propionate and acetate, respectively. Acetate was added at 7mM in the condition inositol-acetate. Mean values are shown with the standard deviation based on 24 h growth experiments performed in duplicate (n=2).

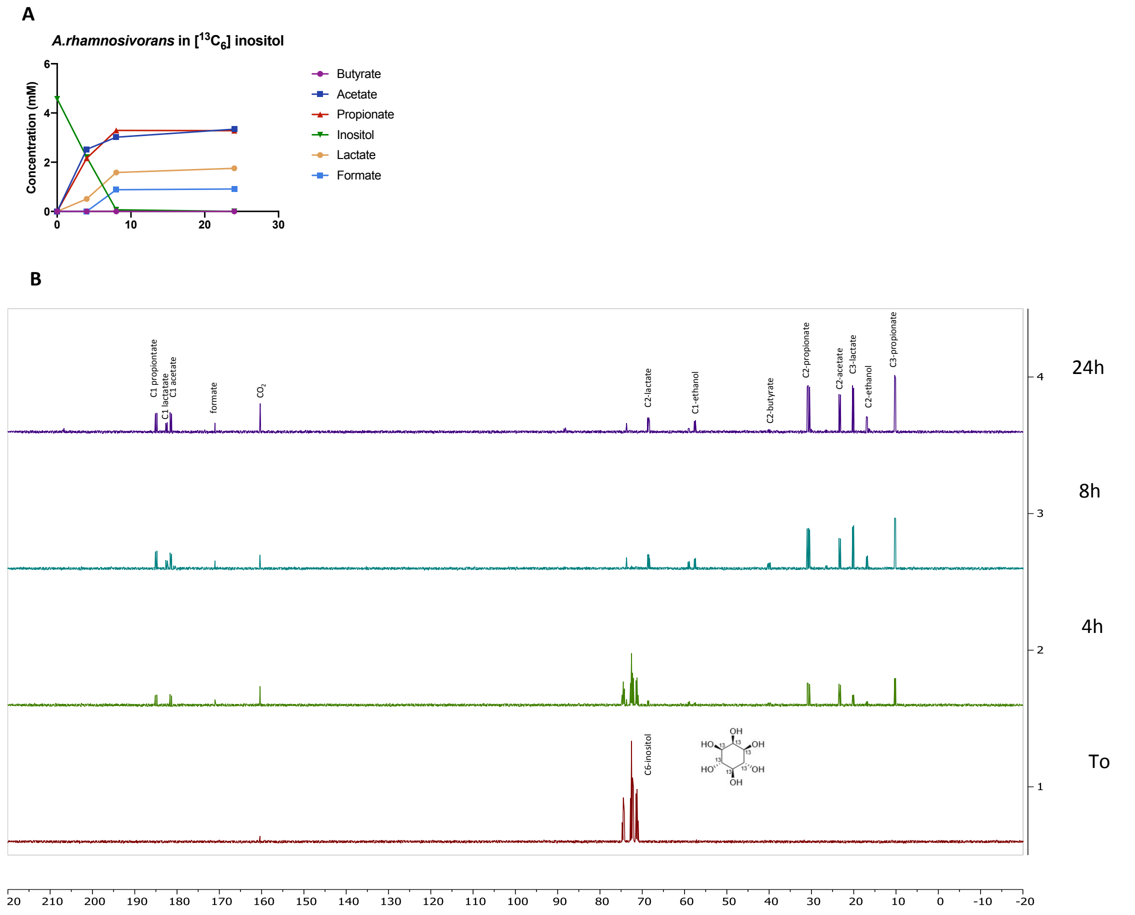

Supplementary Figure 3: Metabolite production from in [$^{13}C_6$]*myo*-inositol by *A. rhamnosivorans*. **A** shows concentrations of substrate and end metabolites analysed using high performance liquid chromatography when the cells were grown in [$^{13}C_6$]*myo*-inositol. **B.** Detection of [^{13}C-labeled end products of [$^{13}C_6$]*myo*-inositol fermentation by *A. rhamnosivorans* during the growth. Supernatants were collected at inoculation (To), 4h, 8h and 24h and used for analyzing the labeled metabolites. [$^{13}C_3$]propionate; [$^{13}C_2$]acetate; [^{13}C]CO_2; [^{13}C]formate; [$^{13}C_3$]lactate; [$^{13}C_2$]ethanol and [2-^{13}C]butyrate were detected as end metabolites. Double peaks indicate the label at multiple carbon atoms in one compound.

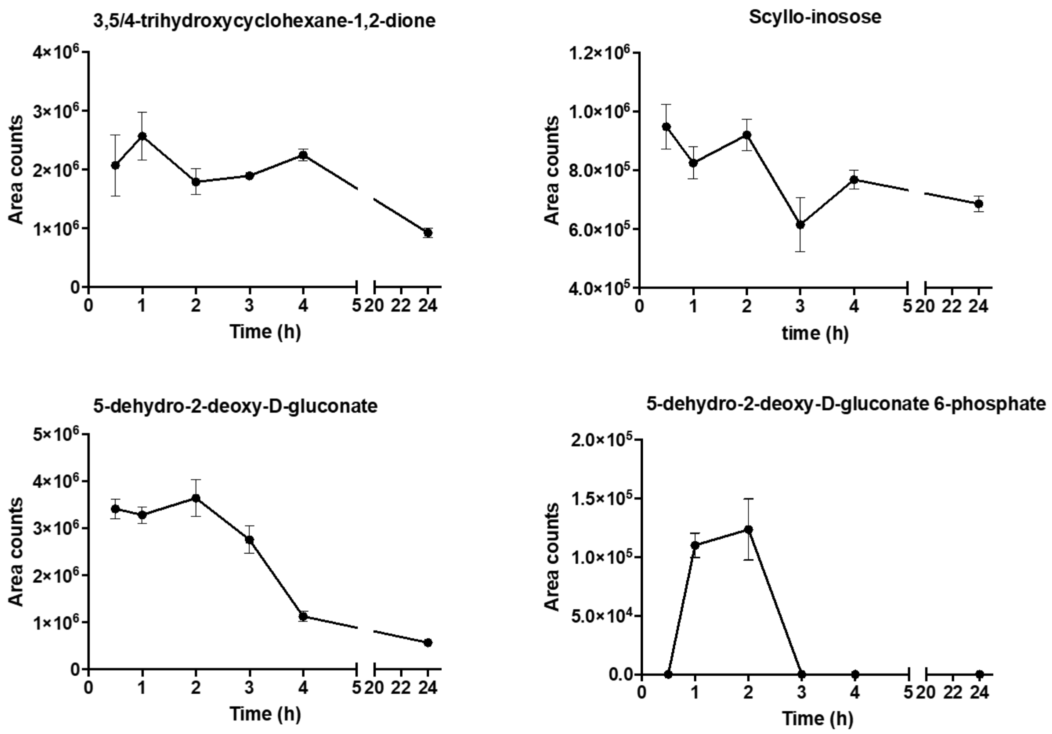

Supplementary Figure 4: Kinetics of *myo*-inositol degradation pathway intermediates monitored by LC-MS/MS over 24 h. Values are reported as area counts. Targeted compounds identification includes profile of scyllo-inosose; 3,5-(4)-trihydroxycyclohexane-1,2-dione; 5-dehydro-2-deoxy-D-gluconate; 5-dehydro-2-deoxy-D-gluconate-6-phosphate. LC-MS/MS analytics are reported in Supplementary Table 4. The measurements were performed in 4 replicates (n=4). Data are presented as mean values +/- SD.

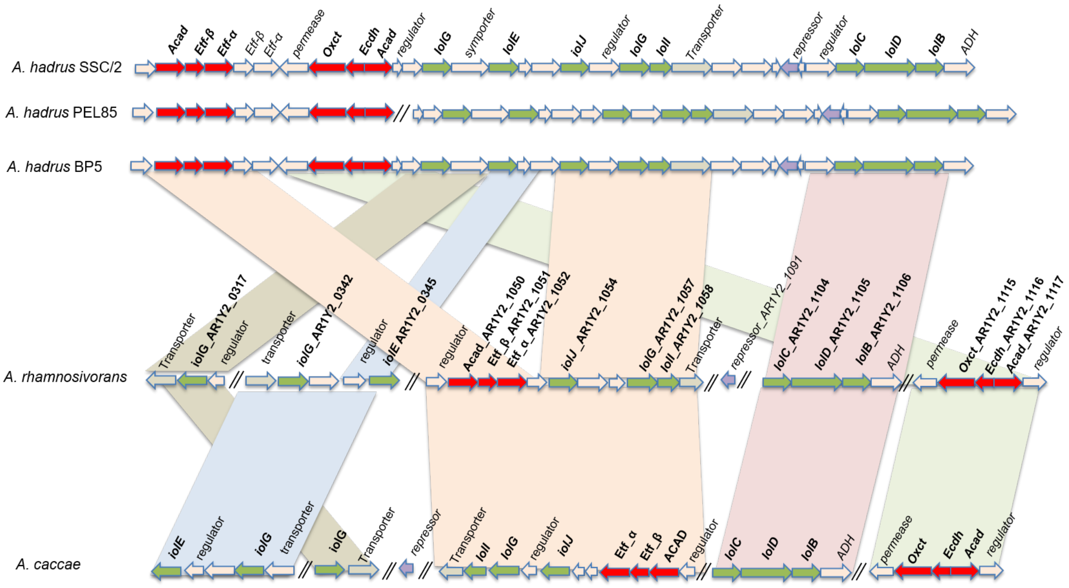

Supplementary Figure 5. *Myo*-inositol pathway gene localization in *Anaerostipes* strains. Architecture of genes involved in *myo*-inositol utilization (green) and those involved in propionate production (red). //: indicates more than one gene in between. Same color shades indicate same gene clusters. The putative transporter gene (with locus tag AR1Y2_1059 in *A. rhamnosivorans)* is indicated in light green

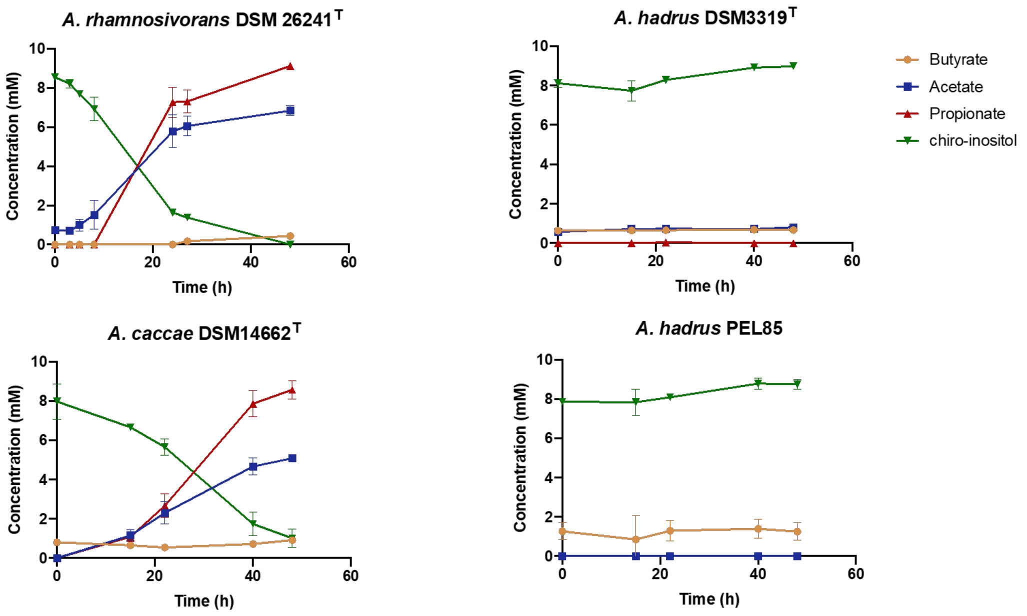

Supplementary Figure 6: *Chiro*-inositol conversion by *A. rhamnosivorans* DSM26241^{T}, *A. caccae* DSM14662^{T}, *A. hadrus* DSM3319^{T} and *A. hadrus* PEL85. The experiment was performed in duplicate. Mean values are shown with the standard deviation. The experiment was performed in duplicate. Data are presented as means +/- SD.

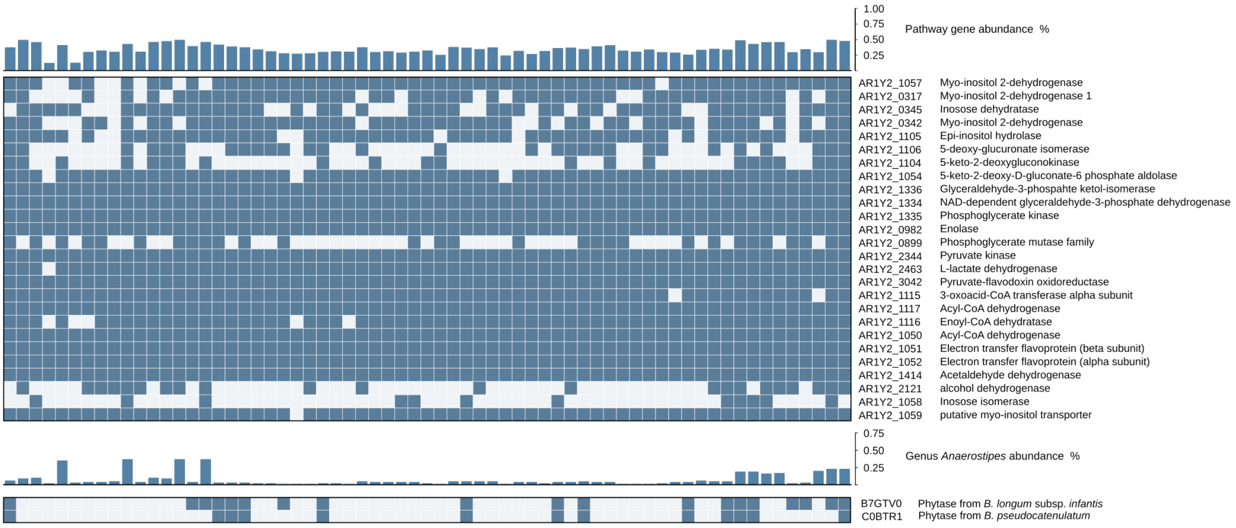

Supplementary Figure 7: Overview of inositol pathway gene and phytase presence in Human Microbiome Project data. Inositol pathway protein absence/presence of at least 1 read in the samples the metagenomics samples with an identity of 40%. Known phytases absence/presence in *Bifidobacterium longum* subsp*. infantis* and *pseudocatenulatum* least 1 read in the samples the metagenomics samples with an identity of 40%. Genome abundances of *Anaerostipes* are shown in each sample.

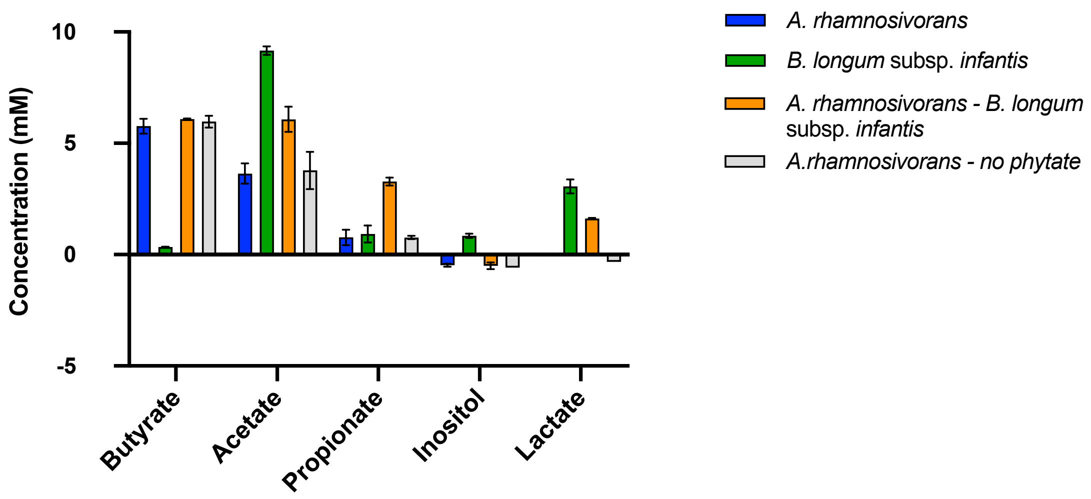

Supplementary Figure 8: Metabolite production from phytate in monocultures of *A. rhamnosivorans*, *B. longum* subsp*. infantis* DSM20088 and the coculture of *A. rhamnosivorans* and *B. longum* subsp*. infantis* DSM20088. Mean values are shown with the standard deviation. The experiment was performed in duplicate and growth was for 48 hours. Small amounts of butyrate and propionate from monoculture of *B.longum* subsp*. infantis* are likely produced from residues of the medium. The production of butyrate in the coculture was likely due to the conversion of carbon sources in yeast extract and peptone in YCFA medium by *A. rhamnosivorans*. Data are presented as mean values +/- SD

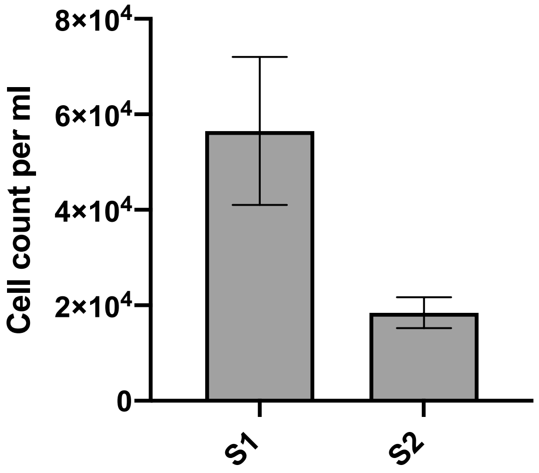

Supplementary Figure 9: Cell numbers of *A. rhamnosivorans* in phytate enrichments from S1 and S2 donors at initial time points by qPCR. The experiment was performed in triplicate. Data are presented as mean values +/- SD.

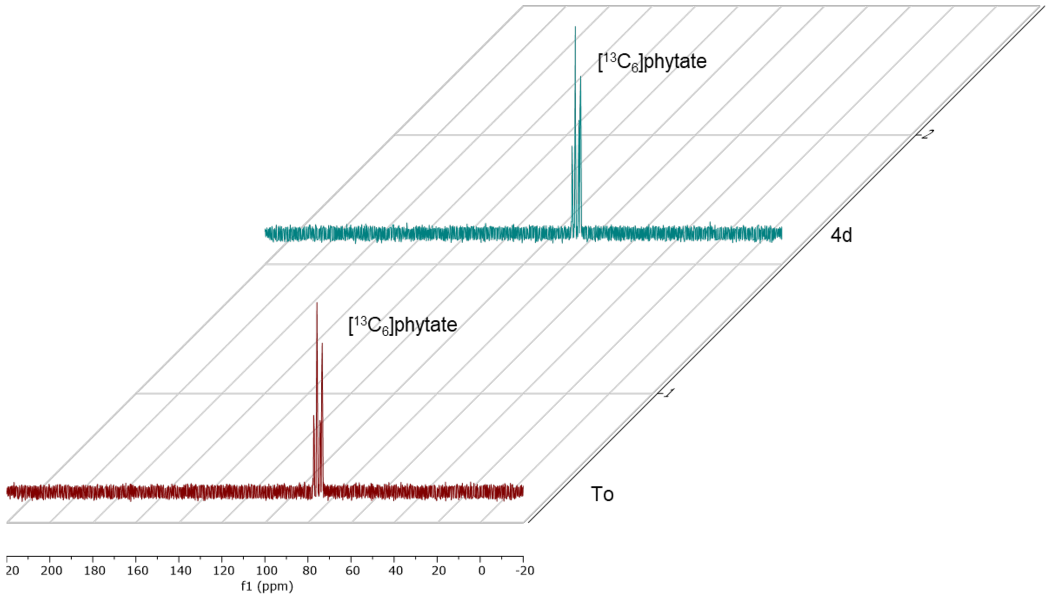

Supplementary Figure 10: Detection of ^{13}C compounds in $^{13}C_6$ phytate fermentation by A*. rhamnosivorans* at initial and end time point. Supernatants were collected at inoculation (To) and 4 days and used for analyzing the labeled metabolites.

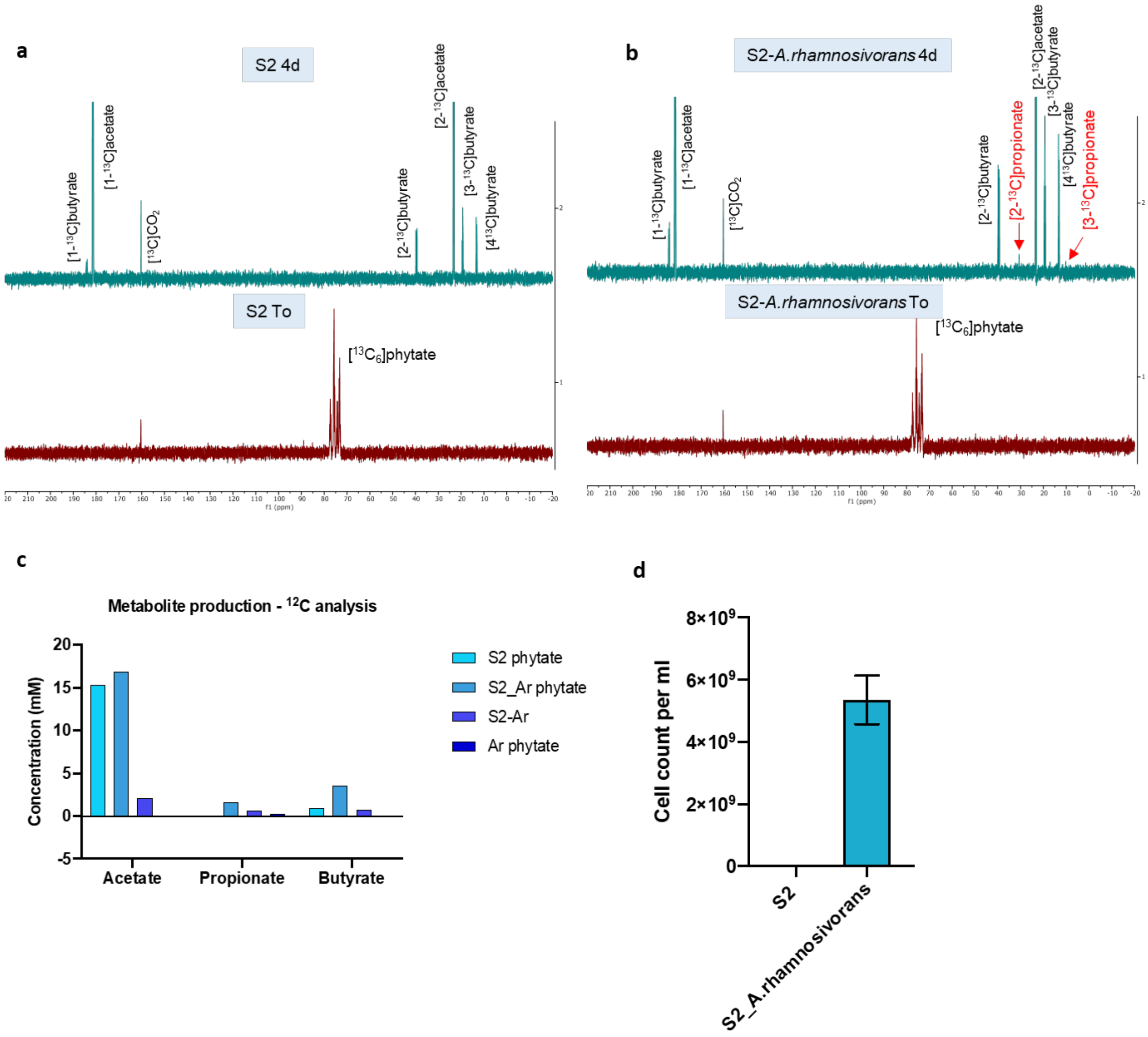

Supplementary Fig. 11: Phytate enrichment by fecal donor 2. Metabolite and substrate analysed by 13C NMR without (A) and with *A. rhamnosivoans* supplementation (B). [^{13}C]propionate was indicated by red arrows in condition *A. rhamnosivorans* was added. Metabolite production was quantified by HPLC analysis (C) and cell counts for *A. rhamnosivorans* by qPCR at initial time point and after 4 day incubation. The qPCR was performed in triplicate (n=3). Data are presented as mean values +/- SD.

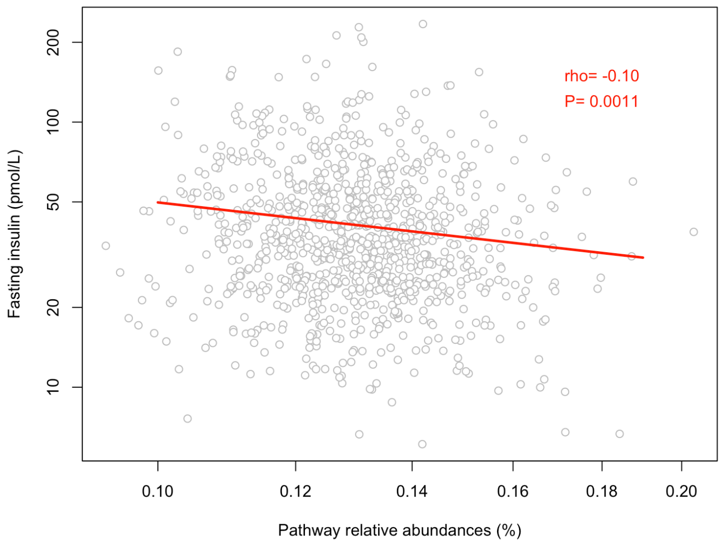

Supplementary Figure 12: Association between the aggregated relative abundances of both cluster 1 and 2 genes involved in inositol metabolism and fasting insulin in the IGT cohort. The rho coefficient and P value were obtained by Spearman correlation. Two-sided statistical tests were used for the analysis.

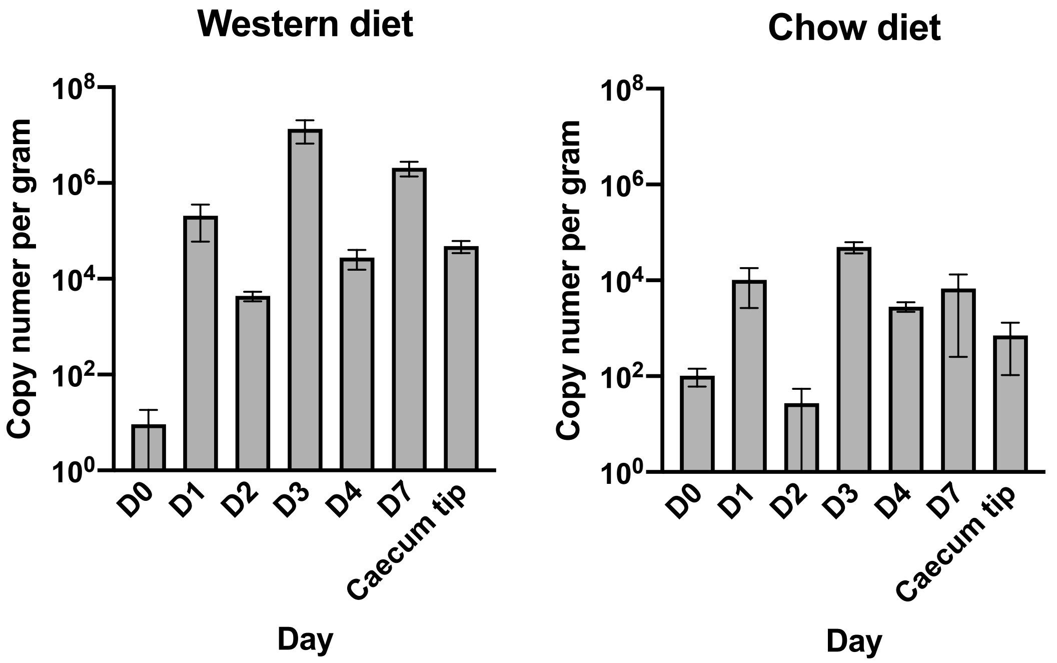

Supplementary Figure 13: *A. rhamnosivorans* quantification by qPCR in western diet fed mice or chow diet fed mice supplemented with *A. rhamnosivorans* (10e9 cell/dose). Stool samples were collected daily and after gavage on the days that the bacteria were administered on day 1 (D1); day 3 (D3) and day 5. Values are means of 5 western diet fed mice (n=5) and 3 chow diet fed mice (n=3). Data are presented as mean values +/- SD.

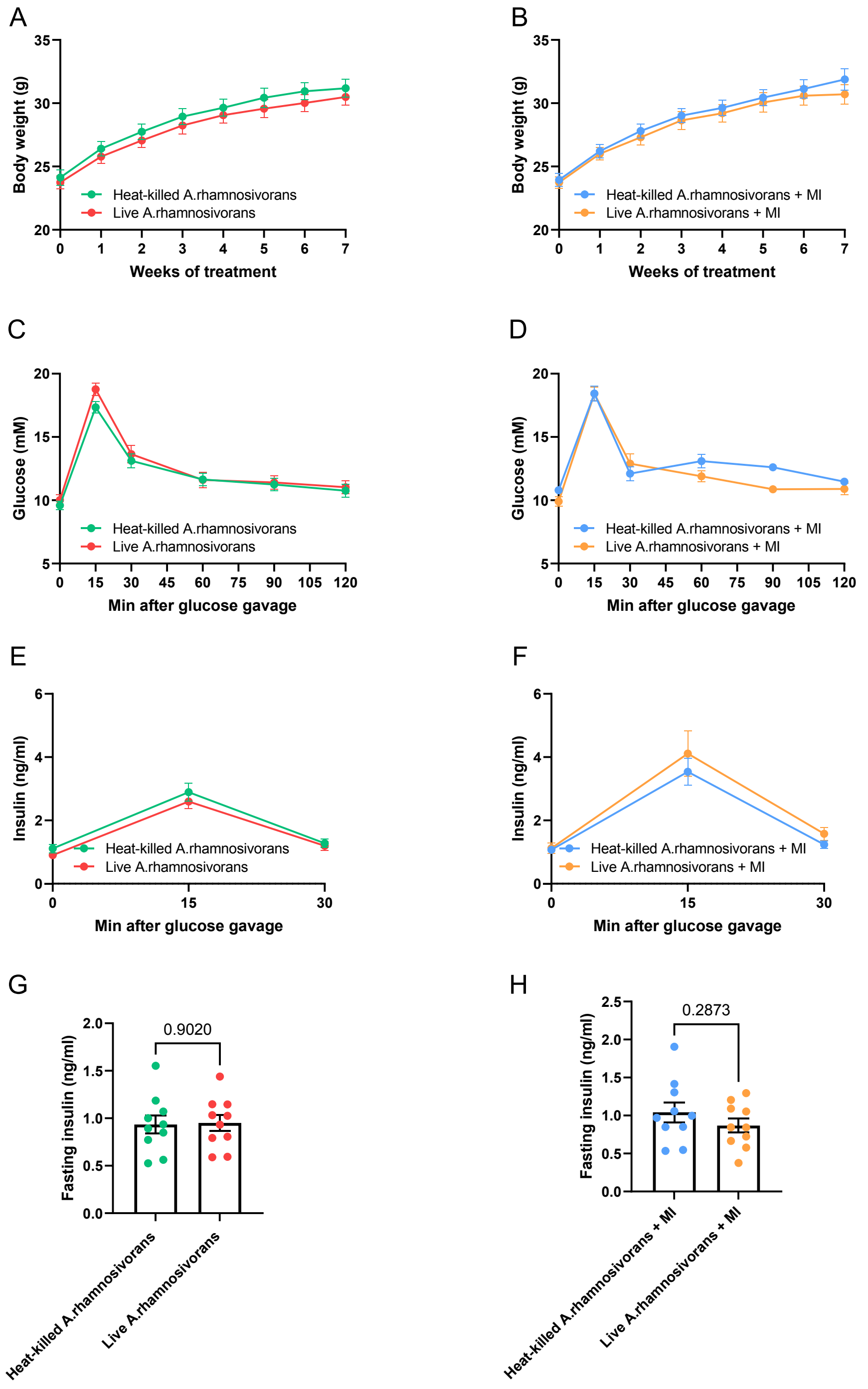

Supplementary Figure 14: Body weight and glucose tolerance test in mice supplemented with live or heat-inactivated *A. rhamnosivorans* in the absence or presence of *myo*-inositol. Body weight was measured once weekly (A-B). Oral glucose tolerance test was performed after 6 weeks of treatment (C-F). Data are presented as mean values ± SEM. n= 10 mice in each group, except for D and F where n=9 for Heat-killed *A. rhamnosivorans* + MI. Unpaired t-test was used for statistical comparison in figure G and H.

Supplementary Table 1. Proteins involved in the *myo*-inositol fermentation pathway and their relative abundance in *myo*-inositol versus rhamnose condition. The fold induction was deduced from the proteome analysis of *A. rhamnosivorans* cells grown on *myo*-inositol and rhamnose. iBAQ values represent the intensity based absolute quantitation. The total intensity was corrected for the number of measurable peptides.

			LogiBAQ (intensity)		
**Locus tag**	**Gene**	**Function**	**Inositol**	**Rhamnose**	**Fold change**
AR1Y2_0316		Tranporter	9,5	7,1	217,4
AR1Y2_0317	*iolG*	Myo-inositol 2-dehydrogenase 1 (EC 1.1.1.18)	11,3	9,2	106,2
AR1Y2_0342	*iolG*	Myo-inositol 2-dehydrogenase (EC 1.1.1.18)	9,5	5,9	4430,1
AR1Y2_0345	*iolE*	Inosose dehydratase (EC 4.2.1.44)	10,7	8,7	98,6
AR1Y2_1104	*iolC*	5-keto-2-deoxygluconokinase (EC 2.7.1.92)	10,8	9,1	51,2
AR1Y2_1105	*iolD*	Epi-inositol hydrolase (EC 3.7.1.-)	11,2	9,6	39,7
AR1Y2_1106	*iolB*	5-deoxy-glucuronate isomerase (EC 5.3.1.-)	10,3	8,6	57,0
AR1Y2_1054	*iolJ*	5-keto-2-deoxy-D-gluconate-6 phosphate aldolase (EC 4.1.2.29)	10,3	9,0	24,5
AR1Y2_1057	*iolG*	Myo-inositol 2-dehydrogenase (EC 1.1.1.18)	9,9	9,9	1,1
AR1Y2_1058	*iolJ*	Inosose isomerase (EC 5.3.99.-)	9,6	9,5	1,1
AR1Y2_1091	*iolR*	Transcriptional repressor of the myo-inositol catabolic operon DeoR family	9,2	9,0	1,4
AR1Y2_1050	*acad*	Acyl-CoA dehydrogenase (EC 1.3.99.2)	10,9	7,2	4813,6
AR1Y2_1051	*etf-β*	Electron transfer flavoprotein, beta subunit	10,6	7,3	1882,1
AR1Y2_1052	*etf-α*	Electron transfer flavoprotein, alpha subunit	10,7	6,9	6440,5
**AR1Y2_1115**	***oxct***	**3-oxoacid CoA transferase**	**10,6**	**7,3**	**2294,1**
AR1Y2_1116	*ecdh*	Enoyl-CoA dehydratase (EC 4.2.1.55)	10,5	8,1	251,7
AR1Y2_1117	*acad*	Acyl-CoA dehydrogenase (EC 1.3.99.2)	10,8	8,3	320,0
		**Shared steps between inositol and rhamnose pathway**			
AR1Y2_3042	*por*	Pyruvate-flavodoxin oxidoreductase (EC 1.2.7.-)	11,4	11,4	0,9
AR1Y2_1334	*gapdh*	NAD-dependent glyceraldehyde-3-phosphate dehydrogenase (EC 1.2.1.12)	10,9	11,0	0,8
AR1Y2_1335	*pgk*	Phosphoglycerate kinase (EC 2.7.2.3)	10,6	10,8	0,7
AR1Y2_1336	*tpi*	Glyceraldehyde-3-phospahte ketol-isomerase (EC 5.3.1.1)	10,2	10,5	0,5
AR1Y2_1339	*pgm*	2,3-bisphosphoglycerate-independent phosphoglycerate mutase (EC 5.4.2.1)	10,1	10,1	1,0
AR1Y2_0982	*enol*	Enolase (EC 4.2.1.11)	10,0	10,1	0,9
AR1Y2_2344	*pk*	Pyruvate kinase (EC 2.7.1.40)	10,5	10,5	1,0
AR1Y2_0899	*pgam*	Phosphoglycerate mutase family (Rhiz)	8,2	7,9	1,6

Supplementary Table 2. Proteins involved in the rhamnose fermentation pathway and their relative abundance in rhamnose versus *myo*-inositol condition. The fold induction was deduced from the proteome analysis of *A. rhamnosivorans* cells grown on myo-inositol and rhamnose. iBAQ values represent the intensity based absolute quantitation. The total intensity was corrected for the number of measurable peptides.

			LogiBAQ (intensity)		Fold
**Locus tag**	**Gene**	**Function**	**Inositol**	**Rhamnose**	**change**
AR1Y2_2131	*L-ri*	L-rhamnose isomerase (EC 5.3.1.14)	7,89	11,00	1280
AR1Y2_2130	*rhaD*	Rhamnulose-1-phosphate aldolase (EC 4.1.2.19)	6,82	10,63	6497
AR1Y2_2132	*rhaB*	Rhamnulokinase (EC 2.7.1.5)	7,37	10,28	812
AR1Y2_2133	*rhaM*	L-rhamnose mutarotase	5,90	8,88	945
AR1Y2_2134	*rhp*	Permease	5,90	9,49	3855
AR1Y2_0175	*nadph*	Lactaldehyde reductase (EC 1.1.1.77)	7,76	10,06	202
AR1Y2_2204	*but*	butyryl-CoA:acetate CoA transferase	10,2	10,4	0,6
		**Shared steps between inositol and rhamnose pathway**			
AR1Y2_3042	*por*	Pyruvate-flavodoxin oxidoreductase (EC 1.2.7.-)	11,4	11,4	0,9
AR1Y2_1334	*gapdh*	NAD-dependent glyceraldehyde-3-phosphate dehydrogenase (EC 1.2.1.12)	10,9	11,0	0,8
AR1Y2_1335	*pgk*	Phosphoglycerate kinase (EC 2.7.2.3)	10,6	10,8	0,7
AR1Y2_1336	*tpi*	Glyceraldehyde-3-phospahte ketol-isomerase (EC 5.3.1.1)	10,2	10,5	0,5
AR1Y2_1339	*pgm*	2,3-bisphosphoglycerate-independent phosphoglycerate mutase (EC 5.4.2.1)	10,1	10,1	1,0
AR1Y2_0982	*enol*	Enolase (EC 4.2.1.11)	10,0	10,1	0,9
AR1Y2_2344	*pk*	Pyruvate kinase (EC 2.7.1.40)	10,5	10,5	1,0
AR1Y2_0899	*pgam*	Phosphoglycerate mutase family (Rhiz)	8,2	7,9	1,6

Supplementary Table 3: Genome comparison using average nucleotide identity (ANI). Calculation of Average Nucleotide Identity (%) of Anaerostipes genomes was performed using JSpeciesWS(http://jspecies.ribohost.com/jspeciesws/#home).

	A. hadrus SSC-2	*A. hadrus* BPB5	*A. hadrus* PEL85	*A. hadrus* DSM 3319	*A. caccae* DSM 14662	*A. rhamnosivorans* DSM 26241
A.hadrus SSC-2	*	97.89	98.05	97.67	69.78	70.01
A.hadrus BPB5	97.55	*	97.66	97.50	70.42	70.52
A.hadrus PEL85	97.74	97.44	*	97.57	69.90	70.01
A.hadrus DSM 3319	98.36	98.07	98.20	*	69.81	69.93
A.caccae DSM 14662	70.24	70.25	70.18	70.05	*	80.47
A. rhamnosivorans DSM 26241	70.21	70.13	70.11	69.91	80.34	*

Supplementary Table 4: Zwitterionic HILIC Fourier transform mass spectrometry (FTMS) analysis. Error (Δ ppm) was calculated as the ratio between the difference of the theoretical mass minus the experimental mass and the theoretical mass, multiplied per one million. Theoretical mass (*m/z* T), experimental mass (*m/z* E), retention time (RT, min), elemental composition (EC). MSI level indicates metabolite identification levels as defined by Metabolomics Standards Initiative (MSI) according to Sumner et al. 2007 [1]

**Compound Name**	**RT**	**EC**	***m/z T***	***m/z EX***	D ppm	**MSI Level**
scyllo-inosose	3.2	C6H10O6	177.04046	177.04031	-0.8	2
3,5/4-trihydroxycyclohexane-1,2-dione	3.1	C6H8O5	159.02989	159.02999	0.6	3
5-dehydro-2-deoxy-D-gluconate	3.5	C6H10O6	177.04046	177.04019	-1.5	2
5-dehydro-2-deoxy-D-gluconate 6-phosphate	4.8	C6H11O9P	257.00679	257.00701	0.9	3

Supplementary references

1. Sumner, L. W.; Amberg, A.; Barrett, D.; Beale, M. H.; Beger, R.; Daykin, C. A.; Fan, T. W. M.; Fiehn, O.; Goodacre, R.; Griffin, J. L.; Hankemeier, T.; Hardy, N.; Harnly, J.; Higashi, R.; Kopka, J.; Lane, A. N.; Lindon, J. C.; Marriott, P.; Nicholls, A. W.; Reily, M. D.; Thaden, J. J.; Viant, M. R., Proposed minimum reporting standards for chemical analysis Chemical Analysis Working Group (CAWG) Metabolomics Standards Initiative (MSI). *Metabolomics* **2007,** *3* (3), 211-221.
