# Supplementary material for: Conversion of dietary inositol into propionate and acetate by commensal Anaerostipes associates with host health
Source: Nat Commun. 2021 Aug 10;12:4798. doi: 10.1038/s41467-021-25081-w (PMC8355322; doi:10.1038/s41467-021-25081-w)
Supplement: Supplementary file 3 — Description of Additional Supplementary Files [file 41467_2021_25081_MOESM3_ESM.docx]

Description of Additional Supplementary Files

Title: Supplementary data 1

Description: List of bacterial species of which NCBI genomes contain cluster 1 (inositol utilization genes) or cluster 2 (propionate production genes) of inositol pathway. Strains marked in red were found to contain both cluster 1 and cluster 2 in their genomes.

Title: Supplementary data 2

Description: Proteomic analysis of *A.rhamnosivorans* DSM26241^T^ grown in *myo*-inositol or rhamnose. The annotated gene function (with locus tags) and the fold induction at the protein level are provided as growth on *myo*-inositol versus growth on rhamnose. Positive values indicate an increase while negative ones indicate a decrease during growth on *myo*-inositol. iBAQ values represent the intensity based absolute quantitation. The total intensity was corrected for the number of measurable peptides. The strain *A.rhamnosivorans* DSM26241^T^ was grown in a bicarbonate buffered medium containing either 40mM *myo*-inositol or 40mM rhamnose. The cells were collected at mid log phase for protein extraction.
