# Supplementary material for: Conversion of dietary inositol into propionate and acetate by commensal Anaerostipes associates with host health
Source: Nat Commun. 2021 Aug 10;12:4798. doi: 10.1038/s41467-021-25081-w (PMC8355322; doi:10.1038/s41467-021-25081-w)
Supplement: Supplementary file 6 — Reporting Summary [file 41467_2021_25081_MOESM6_ESM.pdf]

nature research

Corresponding author(s): Willem M. de Vos; Thi Phuong Nam Bui

Last updated by author(s): 09072021

Reporting Summary

Nature Research wishes to improve the reproducibility of the work that we publish. This form provides structure for consistency and transparency in reporting. For further information on Nature Research policies, see our Editorial Policies and the Editorial Policy Checklist.

Statistics

For all statistical analyses, confirm that the following items are present in the figure legend, table legend, main text, or Methods section.

n/a	Confirmed	
☐	☒	The exact sample size (*n*) for each experimental group/condition, given as a discrete number and unit of measurement
☐	☒	A statement on whether measurements were taken from distinct samples or whether the same sample was measured repeatedly
☐	☒	The statistical test(s) used AND whether they are one- or two-sided<br>*Only common tests should be described solely by name; describe more complex techniques in the Methods section.*
☐	☒	A description of all covariates tested
☐	☒	A description of any assumptions or corrections, such as tests of normality and adjustment for multiple comparisons
☐	☒	A full description of the statistical parameters including central tendency (e.g. means) or other basic estimates (e.g. regression coefficient) AND variation (e.g. standard deviation) or associated estimates of uncertainty (e.g. confidence intervals)
☐	☒	For null hypothesis testing, the test statistic (e.g. *F*, *t*, *r*) with confidence intervals, effect sizes, degrees of freedom and *P* value noted<br>*Give P values as exact values whenever suitable.*
☒	☐	For Bayesian analysis, information on the choice of priors and Markov chain Monte Carlo settings
☒	☐	For hierarchical and complex designs, identification of the appropriate level for tests and full reporting of outcomes
☐	☒	Estimates of effect sizes (e.g. Cohen's *d*, Pearson's *r*), indicating how they were calculated

Our web collection on statistics for biologists contains articles on many of the points above.

Software and code

Policy information about availability of computer code

Data collection	Amino acid sequences were obtained on National Center for Biotechnology Information (NCBI) with provided accession numbers. NCBI reference genomes were used to screen bacteria with presence of complete inositol pathway.
Data analysis	HPLC spectra were obtained using Chromeleon7 (ThermoFisher Scientific); Hydorgen was measured in a compact GC and visualized by Chromeleon console (ThermoFisher Scientific). NMR spectra were obtained using Brucker TopSpin 3.5 (Brucker). NMR plots was made using MestRenova 14.1 (Mestrelab Research SL). MS and MSMS spectra were acquired using Xcalibur 4.2.47 and all spectra were analyzed with MaxQuant 1.6.3.4 (Max-Planck-Institute of Biochemistry, Germany). Genome annotation was performed on RAST server (rapid Annotations using Subsystems Technology). Pairwise genome comparison was done on JSpeciesWS server (Ribocon). The heatmap was made with seaborn python. Phylogenetic tree was made using Clustal X2 (version 2.1; Conway Institue UCD Dublin); Mega7 (version 7.0.26; Megasoftware). For metagenomic analyses; read alignment of fasta sequences against the pathway proteins was performed with Diamond (v0.9.22); Heatmap was made with Python3.8.2; Kraken2 (2.0.9-beta) was used for taxonomic read classification in the metagenomic samples; abundance estimation of the genus Anaerostipes (taxonomy ID 207244) was determined by Bracken (v2.5). Enzyme assays were measured on Hitachi spectrophotometer with UV solutions software (2007). All figures were made using Prism 8 (GraphPad). The KO relative abundances in (pre)diabetic cohorts were associated with measured clinical biomarkers based on Spearman correlation analysis (P<0.1; *, P<0.05; +, P<0.01; #, P<0.001). Animal study: Statistical analysis was done by Unpaired two-tailed Student's t tests. Pearson correlation (E) between cecal propionate:butyrate ratio and fasting insulin in mice treated with heat-killed or live A. rhamnosivorans plus myo-inositol.

For manuscripts utilizing custom algorithms or software that are central to the research but not yet described in published literature, software must be made available to editors and reviewers. We strongly encourage code deposition in a community repository (e.g. GitHub). See the Nature Research guidelines for submitting code & software for further information.

Data

Policy information about availability of data

All manuscripts must include a data availability statement. This statement should provide the following information, where applicable:

- Accession codes, unique identifiers, or web links for publicly available datasets
- A list of figures that have associated raw data
- A description of any restrictions on data availability

The whole-genome sequence of A. rhamnosivorans DSM26241T has been deposited in NCBI under the BioProject accession number PRJNA540423 and SRA accession number CP040058. The raw mass spectrometry proteomics data have been deposited to the ProteomeXchange Consortium via the PRIDE repository with the dataset identifier PXD021084 (https://www.ebi.ac.uk/pride/archive/projects/PXD021084). The entire proteome is provided in the Supplementary information/ source data file. Metagenomes of Swedish Impaired Glucose Tolerance (IGT) cohort were used for pathway analyses (DOI: 10.1016/j.cmet.2020.06.011). The clinical data are available under restricted access for ethical and legal considerations, access can be obtained following the standard application to the data committee. Other associated data generated in this study are provided in the Supplementary Information/Source Data file. The raw metagenomics data used in this study are available in the China NGDC Genome Sequence Archive under accession code HRA000020 [https://ngdc.cncb.ac.cn/gsa-human/browse/HRA000020]. Used databases are biological Magnetic Resonance Data bank (http://www.bmrb.wisc.edu/metabolomics/metabolomics_standards) ; Pfam (http://pfam.xfam.org/), InterPro (https://www.ebi.ac.uk/interpro/), Brenda (https://www.brenda-enzymes.org/), Uniprot (https://www.uniprot.org/); NCBI genome (https://www.ncbi.nlm.nih.gov/genome/). Human Metabolome Database (https://hmdb.ca/), KEGG (https://www.genome.jp/kegg/compound/). Source data are provided with this paper.

Field-specific reporting

Please select the one below that is the best fit for your research. If you are not sure, read the appropriate sections before making your selection.

☒ Life sciences ☐ Behavioural & social sciences ☐ Ecological, evolutionary & environmental sciences

For a reference copy of the document with all sections, see nature.com/documents/nr-reporting-summary-flat.pdf

Life sciences study design

All studies must disclose on these points even when the disclosure is negative.

Sample size	The growth experiment on myo-inositol were performed for all publicly available Anaerostipes strains (6x). To further elucidate the pathway, Anaerostipes rhamnosivorans was used as model organism. The metagenomic analysis was performed using a publicly available dataset that contains 65 subjects from the Human Microbiome Project. The association between inositol pathway genes and measured clinical biomarkers based on Spearman correlation was performed in Swedish prediabetic cohorts of 1495 subjects. This sample size of the cohort was determined according to power calculation (85% power) based on the incidence of T2D from IGT as previously shown (Fagerberg B et al. Adiponectin in relation to insulin sensitivity and insulin secretion in the development of type 2 diabetes: a prospective study in 64-year-old women. J Intern Med. 2011). Entire NCBI reference genomes release 92 (around 10 000 genomes) were used to screen bacteria with the presence of entire inositol pathway. The animal study was performed with 4 different treatment groups with 10 mice per group. This results in an E-value of 36 (E = Total number of animals − Total number of groups), which is considered to be more than acceptable (E>20).
Data exclusions	No data were excluded
Replication	The experiment was performed in triplicate for growth experiments; in 4 replicates for proteomics; in technical triplicate and biological duplicate for enzyme assays. LC-MS/MS were performed with biological duplicates. qPCR was performed in triplicate.The 13C- NMR was performed with one replicate for all labeled compounds due to the limited 13C resources and all measurements were successfully done that have been included in the manuscript. All attempts at replication were successful.
Randomization	Before treatment start the investigator randomized the animals into to 4 treatment groups and made sure the groups did not differ in body weight. The animal arrived with 5 mice in each cage and these cage setups cannot be changed since males will start to fight.
Blinding	Blinding was not possible since the investigator needed to treat each animal with the correct substance for the designated group.

Reporting for specific materials, systems and methods

We require information from authors about some types of materials, experimental systems and methods used in many studies. Here, indicate whether each material, system or method listed is relevant to your study. If you are not sure if a list item applies to your research, read the appropriate section before selecting a response.

Materials & experimental systems

n/a	Involved in the study
☒	☐ Antibodies
☒	☐ Eukaryotic cell lines
☒	☐ Palaeontology and archaeology
☐	☒ Animals and other organisms
☐	☒ Human research participants
☒	☐ Clinical data
☒	☐ Dual use research of concern

Methods

n/a	Involved in the study
☒	☐ ChIP-seq
☒	☐ Flow cytometry
☒	☐ MRI-based neuroimaging

Animals and other organisms

Policy information about studies involving animals; ARRIVE guidelines recommended for reporting animal research

Laboratory animals	All animal experiment were done in male C57Bl/6J mice (JAX™ Mice Strain, Charles River Laboratories, Germany)
Wild animals	Did not involve wild animals
Field-collected samples	Mice were housed in individually ventilated cages (Green line Sealsafe plus, Tecniplast, Buguggiate, Italy) with a 5 mice per cage. Mice were kept under constant temperature and a 12-h light/dark cycle with free access to food and water.
Ethics oversight	All animal experiments were conducted in accordance with the principles of the 'Guide to the Care and Use of Experimental Animals' and were approved by the Ethics Committee on Animal Care and Use in Gothenburg, Sweden

Note that full information on the approval of the study protocol must also be provided in the manuscript.

Human research participants

Policy information about studies involving human research participants

Population characteristics	Stool samples of 2 healthy aldults were used for phytate enrichment experiment.
Recruitment	2 healthy aldults without any disease background were randomly chosen
Ethics oversight	Fresh stools were collected from 2 healthy donors of whom informed consents were obtained following Good Clinical Practice. We have complied with all relevant ethical regulations for work with human participants.

Note that full information on the approval of the study protocol must also be provided in the manuscript.
